# Investigation of the Immunomodulatory effect of *Berberis vulgaris* on core-pulsed dendritic cell vaccine

**DOI:** 10.1186/s12906-016-1327-2

**Published:** 2016-08-30

**Authors:** Doaa A. Ghareeb, Eiman H. Elwakeel, Rowaida Khalil, Mina S. Aziz, Maha A. El demellawy

**Affiliations:** 1 Biochemistry Department, Faculty of Science, Alexandria University, Alexandria, Egypt; 2 Center for Aging and Associated Diseases, Helmy Institute for Medical Sciences, Zewail City, Egypt; 3 Botany and Microbiology Department, Faculty of Science, Alexandria University, Alexandria, Egypt; 4 Biomedical Technology, GEBRI, SRTA-city, Alexandria, Egypt

**Keywords:** IL 12, *Berberis vulgaris*, Th-1, IL 4, INF-γ

## Abstract

**Background:**

Virus-induced dendritic cells (DCs) functional deficiency leads to sub-optimal initiation of adaptive immune responses and consequently chronic infection establishment. The present study reports an advanced hepatitis C virus (HCV) therapeutic vaccine model based on In vivo enrichment of DCs with barberry ethanolic crude extract (BCE) then pulsing them with HCV core protein.

**Methods:**

DCs were enriched by BCE intravenous injection in BALB/c mice. Vaccine efficiency was assessed by flow cytometric analysis of splenocytes of immunized mice, cytokine profiling, cytotoxic T lymphocyte assay, and humoral immune response assessment.

**Results:**

There was no significant difference in surface phenotypic characterization of splenocytes from mice immunized with non-BCE-enriched-core-pulsed DCs (iDcs-core) compared to those from mice injected with RPMI-1640 medium. However, splenocytes from mice immunized with BCE-enriched-core-pulsed DCs showed 197 % increase in CD16+ population, 33 % increase in MHCII^+^ population, and 43 % decrease in CD3^+^ population. In iDCs-core group, 57.9 % greater anti-core cytotoxic T lymphocyte activity, up-regulation in interferon gamma and interleukin (IL) -12 expression, and down-regulation in IL-4 and IL-10 were recorded. Moreover, sustained specific anti-core antibodies were detected only in sera of the same group.

**Conclusions:**

results indicate that BCE-enriched-core-transduced DCs may serve as a new model for immunotherapy of HCV chronic infection.

## Background

Approximately 180 million people worldwide are chronically infected with HCV, but most are unaware of the infection based on the lack of symptoms at its first stages [1]. The global seroprevalence of anti-HCV antibodies is estimated to be 2.8 %. Central and East Asia and North Africa/Middle East are estimated to have the high viral prevalence >3.5 %; [2] the highest estimated annual number of HCV infections locates in Egypt where there is more than 150,000 new patients every year [3].

Standard of care (SOC) antiviral therapy for HCV today consists of pegylated IFN-α-2a or -2b in combination with ribavirin. Patients with genotype 1 and 4 typically require 48 weeks of SOC treatment with sustained virologic response (SVR) of around 40 % to 50 %, while those with genotype 2 and 3 require 24 weeks of treatment with SVR of around 70 % to 80 % [4].

Development of an effective vaccine against HCV infection has long been defined as a difficult challenge. This is due to the quasispecies nature of the virus due to the lack of proofreading activity of the viral RNA-dependent RNA polymerase, and the observation that convalescent humans and chimpanzees could be re-infected upon re-exposure [5]. HCV chronic infection is characterized by the absence of efficient antiviral T cell responses. Virus-induced defect in DCs function is one of the mechanisms responsible for immune evasion of HCV. [6] A number of studies described a significant down-regulation of DCs function in HCV infected patients, where Th1/Th2 balance was shifted towards Th2 up-regulation [7, 8]. Thus, vaccination strategies to induce strong anti-HCV T cell responses are essential for both of prophylactic and therapeutic purposes.

DCs are of the greatest antigen presenting capacity among all immune effectors; therefore enriching such cells with a natural and potent agent to counteract the defect caused by viral infection is of a major concern. Immunization with DCs loaded with some conserved viral parts offers a new approach for induction of a specific antiviral immunity and developing a novel therapeutic strategy in HCV infection therapeutics [9].

*Berberis vulgaris* L. is well known medicinal plant with traditional herbal medical history. Used in many civilizations as a curative herbal remedy in the homeopathic system of medicine [10]. The most important constituents are isoquinoline alkaloids, such as berberine, berbamine and palmatine [11].

It was demonstrated that treatment of macrophages and DCs with berberine; a benzodioxoloquinolizine alkaloid present in *Berberis vulgaris* plant, significantly induced interleukin (IL) -12 production in a dose-dependent manner, Therefore, it could be counteracting the effect of HCV infection of misbalancing the Th1/Th2 cytokines ratios to evade the immune response of the host [12].

Our published in vitro study showed that the treatment of mice splenocytes with BCE induced interferon gamma (IFN-γ) production and increased the level of CD11c which indicated the positive increment of antigen representing cells specially DCs. Moreover, BCE increased the production of both IFN-g and IL-12 and decreased the production of IL 10 and IL 4 therefore BCE shifted the maturation towards the Th1 [13].

Therefore, therapeutic DCs vaccine may hold its promises by working on DCs maturation and proliferation, since DCs of chronically infected patients are under negative regulation of the virus itself. Immunization strategies in chronic HCV infection may have one central aim in the future: targeting directly or indirectly DCs to induce vigorous immune responses that are able to eliminate the virus. The present study reports an advanced hepatitis C virus (HCV) therapeutic vaccine model based on In-vivo enrichment of DCs with barberry ethanolic crude extract (BCE) and pulsing them with HCV core protein.

## Methods

### BEC extraction and animal modeling

Barberry dried roots were purchased and imported from Iran. They were authenticated by Prof. Dr Salama El- Dareir from the Botany Department, Faculty of Science, Alexandria University, Egypt. First, this classification was determined based on the data about the plant, published in Dragon Herbarium (https://dragonherbarium.com/products/barberry-root-bark-c-s-wc-berberis-vulgaris).

BEC was prepared from *Berberis vulgaris* root according to Abd-Elwahab et al., [14] and Ghareeb et al. [15] where The dried powdery roots of barberry was exhaustively defatted with petroleum ether and subjected to steam distillation method for ethanolic gradient extraction with Soxhlet apparatus. The ethanolic extract was concentrated to minimum volume using rotary evaporator then lyophilized to obtain a powder extract of barberry (25 %). The barberry extract powder form was kept at −20 °C until subjected to further biochemical analysis BALB/c female mice, inbred strain (8–10 weeks of age, 25–30 g body weight) were purchased from experimental animal house (Tudor Belharis Research Institute), and housed in the animal house of Medical Technology Centre, Medical Research Institute, Alexandria University, Egypt. all study protocols for animal and biological tissue samples treatment, involved in this study, were firmly subjected to ethical instructions outlined by Animal Ethics Committees (AEC) that published via The National Health and Medical Research Council (NHMRC) policies and guidelines that recommended by the Egyptian Ministry of Health and Population, High Committee Of Medical Specialties, Arab Republic of Egypt [16]. This study granted permission from the Biomedical technology, SRAT-city and Biochemistry Department, Faculty of Science, Alexandria University, following approval of the Research Ethics Committee, Faculty of Pharmacy, Alexandria University.

Animals were grouped and housed in metal cages (eight mice/cage), maintained at approximately 23–25 °C with a 12 h light/dark cycle and received laboratory basal diet and tap water for 1 week acclimation period throughout the study period. In this study, two successive animal models for DCs enrichment and immunization were used.

### In vivo DCs enrichment, separation and transduction

DCs were enriched by intravenous injection of mice with 60 mg/kg of BEC eight times every other day. Mice were grouped into two groups (8 mice/group); the first was designated as BCE-induced-DCs, while the second which received no treatment was designated as non-BCE-induced-DCs. Two days after the last injection mice were euthanized by cervical dislocation and spleens were dissected and dissociated with cell dissociate (gentleMACS™, Miltenyi Biotec, Germany) for single-cell suspension in RPMI-1640 plain medium (Lonza, USA). Cells were centrifuged at 1650 rpm for 5 min, brake 9/9, at 25 °C. Pellets were dispersed with 10 ml of red blood cells lysis buffer, and centrifuged at the same conditions. Pellets were collected and dispersed again in 1 ml RPMI - 1640 complete medium and cells were counted using 0.4 % trypan blue. DCs were separated from total splenocytes using anti-CD11c microbeads (MACS, Miltenyi Biotec, Germany). Influence of BEC on enriching DCs population was evaluated by staining the separated cells with PE-conjugated hamster anti-mouse CD11c antibody (Miltenyi Biotec, Germany) and counting CD11c^+^ cells using FACS analysis (Becton Dickinson, USA (using CellQuest Pro Software. CD11c^+^ cells were transduced with HCV core recombinant peptides (Sigma Aldrich, USA) at a concentration of 2 μg/10^6^ cells.

### Phenotypic analysis of DCs

After the DCs were collected, the level of CD11c and MHCII were detected by FACSCalibur flow cytometer (BD Biosciences). Moreover, cytokines expression (IL4, IL10, IL 12 and INF-gamma) was performed by RT-PCR beside IL 12 and INF-gamma proteins levels were measured by ELISA (Komabiotech, Korea).

### Invivo immunization

This step required an animal model design of six groups of BALB/c mice (eight mice/group). According to the general aspects of vaccination of world health organization (WHO), primary immunization schedules includes at least two vaccine doses, optimally repeated at a minimal interval of 3–4 weeks to generate successive waves of immune response. Accordingly, mice were treated subcutaneously three times at four-weeks intervals with different DCs subsets obtained from animal model A (ex-vivo transduced with core recombinant protein or non-transduced) resulting in the following groups: 1) Negative control group that received no treatment, 2) RPMI - 1640 group that received complete medium, 3) nDCs group that received 0.1 × 10^6^ non-BCE-induced DCs at the first injection, and 0.05 × 10^6^ of the same cells at the second and third injections. 4) nDCs-core that received 0.1 × 10^6^ non-BCE-induced DCs transduced with core protein at the first injection, and 0.05 × 10^6^ at the second and third injections. 5) iDCs group that received 0.1 × 10^6^ BCE-induced DCs at the first injection, and 0.05 × 10^6^ at the second and third injections. 6) iDCs-core group that received 0.1 × 10^6^ BCE-induced DCs transduced with core protein at the first injection, and 0.05 × 10^6^ at the second and third injections. Immunization scheme was performed according to sakai et al. [17] with modifications as follows: 1) A dose lower than 0.1 × 10^6^ DCs/mouse had been administrated to mice to avoid death with hypoxia, 2) Immunization was performed in four weeks intervals, not three weeks intervals.. After immunization period, mice were euthanized by cervical dislocation at day 7 after the last booster exposure, and spleens were dissected and dissociated as previously mentioned.

### Phenotypic characterization of splenocytes of immunized mice by flow cytometric analysis

Splenocytes were washed and harvested in a 96-well plate. 10 μl of FITC-conjugated monoclonal anti-mouse MHCII (Miltenyi Biotec, Germany), APC-conjugated monoclonal anti-mouse Fcγ RIII/CD16, and PE-conjugated monoclonal anti-mouse CD3 (R&D systems, UK) antibodies were added individually to wells containing 0.1 × 10^6^ cells dispensed in 25 μl PBS, and incubated at 4 °C for 45 min according to the manufacturer’s instructions. After incubation, the unreacted antibody reagent was discarded by washing the cells twice with 4 ml of same 0.5 % BSA in PBS. Cells were re-suspended in 200 μl PBS/BSA buffer for flow cytometric analysis.

### Cytokines profile

Cytokines profile was studied on the genomic level by reverse transcriptase polymerase chain reaction (RT-PCR), and on protein level by enzyme linked immunosorbent assay (ELISA). Total RNA was extracted from immunized splenocytes (10^6^ cells) according to the method of Chomczynski et al. [18] First strand cDNA was synthesized using RevertAid™ first strand cDNA synthesis kit (Fermentas, Thermo fisher scientific, Germany). RT was performed using DreamTaq™ Green PCR master mix (2X) (Fermentas, Thermo fisher scientific, Germany), and cytokine-specific oligonucleotide primers were: GAPDH, 5′-CAAGGTCATCCATGACAACTTTG-3′, 5′-TCCACCACCCTGTTGCTGTAG-3′; INF-γ, 5’-GTCAACAACCCACAGGTCCAG-3′, 5′-TTGGGACAATCTCTTCCCCA-3′; IL-12, 5′-CAGAAGCTAACCATCTCCTGGTTTG-3′, 5′-TCCGGAGTAATTTGGTGCTTCACAC-3′; IL-10, 5′-AGAGACTTGCTCTTGCACTACCAA-3′, 5′-GTAAGAGCAGGCAGCATAGCAGT- 3′; IL-4, 5′-TTGTCTCTCGTCACTGACGCA-3′, 5′-TACGAAGCACCTTGGAAGCC-3′. The amplification was performed at initial step at 95 ° C for 15 min, followed by 40 cycles of a denaturation step at 90 °C for 30 s, an annealing step at primer’s melting point - 5 °C for 1 min, an extension step at 72 °C for 8 min, and a final extension step at 72 °C for 10 min. The resulting PCR fragments were visualized on 1.5 % agarose gels, and gel bands were revealed using gel documentation system. The relative expression quantity was evaluated by the ratios of band intensity to GAPDH using AlphaView software. Cytoplasmic contents of cytokines protein expression of INF-γ was screened according to the instructions of Mouse INF-γ ELISA kit (Komabiotech, Korea).

### Determination of the optimum effector cells: target cells ratio (E:T)

In a 96-well rounded-bottomed plate, splenocytes of non-treated mice were co-cultured with murine EL4 cells (ATCC® TIB39 ™) with a final cell count of 0.5 × 10^6^ cell/well at E: T ratios of 1:25, 1:12.5, 1:6.2, 1:3.1 and 1:1.6. The plate was incubated at 37 °C for 4 h in presence of 5 % CO_2_. 20 μl aliquots of MTT (Sigma-Aldrich, USA) (were added in the dark to each well, and the plate was re-incubated at 37 °C and 5 % CO_2_ for 4 h. The plate was centrifuged at 25 °C, 2000 rpm and brake 9/9 for 10 min. Supernatants were discarded, 200 μl of DMSO was added to each well, and the plate was agitated using a shaker (ThermoFisher) for 5 min. Absorbance was determined at 570 nm on microtiter plate reader (BioTek, USA).

### EL4 cells transduction with core protein

Transduction of 1 × 10^6^ cell of EL4 with 10 μg of core recombinant peptides was performed using protein delivery system BioPORTER quickEase protein delivery Kit (Sigma-Aldrich, USA). Transduction was evaluated using anti-HCV core antigen antibody (Abcam, UK) as described by Coligan et al. [5].

### Cytotoxic T lymphocyte assay by LDH and MTT

Cells (1.0 × 10^6^) were dispensed at an E: T ratio of 1:1.6 into a 96-well rounded plate, brought to 150 μl with complete medium and incubated at 37 °C for 4 h in presence of 5 % CO_2_. Co-cultured cells were centrifuged at 25 °C, 2000 rpm and brake 9/9 for 10 min. LDH release was assessed according to the instructions of Lactate cytotoxicity assay kit (Cayman chemicals, USA). With the same plate set-up, MTT assay was performed as previously described.

### Monitoring humoral response

Anti-HCV core IgG antibody levels) total IgG) in the serum of each immunized mouse were screened against core recombinant peptides and non-structural protein3 (NS3) antigen (Sigma-Aldrich, USA) according to Lazdina et al. [19] Efficiency of anti-core antibodies was estimated using non-structural protein 3 (NS3) as a negative control.

### Statistical analysis

Data were analyzed by one-way analysis of variance (ANOVA) using Primer of Biostatistics (version 5) software program. Significant differences (p < 0.05) between the means ± SD were determined using multiple comparisons Student-Newman-keuls test.

## Results

### Remarkable increase in splenic CD16 and MHCII along with diminished CD3

**Fig. 1:**
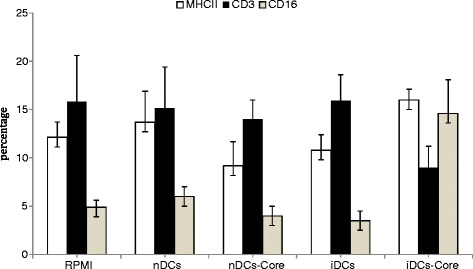
Effect of immunization with different subsets of DCs on surface marker expression in splenocytes of immunized mice. Splenocytes of immunized mice with BCE-induced-core pulsed dendritic cells, in a 3-dose immunization schedule at 4 weeks intervals, shows an elevated CD16^+^and MHCII^+^ populations when compared to RPMI - 1640 group. Spleens of immunized mice were dissociated by gentleMACS dissociator, as described in Materials and Methods, into a single-cell suspension. Freshly isolated splenocytes were stained with MHCII-FITC, Fcγ RIII/CD16-APC, and CD3-PE monoclonal antibodies and analyzed by flow cytometry. Data are shown as the percentage of splenocytes bearing surface antigen over total splenocytes population. * indicates a significant difference with RPMI group. Results are means ± SD of splenocytes of six mice. (PRPMI - 1640 group that received complete medium, nDCs group that received non-BCE-induced DC, nDCs-core group that received non-BCE-induced DCs transduced with core protein, iDCs group that received BCE-induced DCs, iDCs-core group that received BCE-induced DCs transduced with core protein)

A panel of monoclonal antibodies were used to detect CD16, MHCII, CD3 surface antigens individually on splenic DCs for tracking the phenotypic changes of splenocytes after immunization. It has been known that CD16-mediated signal transduction promotes induction of maturation of immature DCs and CD16-mediated antigen uptake potently enhances antigen presentation [20, 21]. Expression of MHCII surface antigen identifies antigen presentation, DCs morphology, and expression of co-stimulatory molecules [22]. While CD3 expression, T-cell co-receptor is a protein complex, reflects the ligation of CD3 with T complex receptor (TCR) induced by MHCII:core recombinant peptide ligation. Because, multiple immune defects had been described in patients with chronic HCV infection that may be linked to the reduced INF-α production, including insufficient response of CTL, low activity of natural killer cells (NK cells), we tested the hypothesis that immunizing mice with DCs pre-stimulated with BCE may have a better antigen presenting capacity and NK activity. Using flow cytometry we found iDCs-core was the only group that showed a significant phenotypic change characterized by the difference in surface antigens expression when compared to RPMI - 1640 group (p < 0.05, n = 6) as follows; 197.9 % increase expression of CD16 surface antigen; 32 % increase expression of MHCII surface antigen; while 43 % decrease expression of CD3 surface antigen (Fig. 1).

### High Th1 cytokines versus low Th2 cytokines

A number of studies recently described a significant down-regulation of DCs function in HCV infected patients, shifting the Th1 (INF-γ and IL-12) /Th2 (IL-4 and IL-10) balance towards Th2 up-regulation [7–15, 17–23] (https://dragonherbarium.com/products/barberry-root-bark-c-s-wc-berberis-vulgaris).

**Fig. 2:**
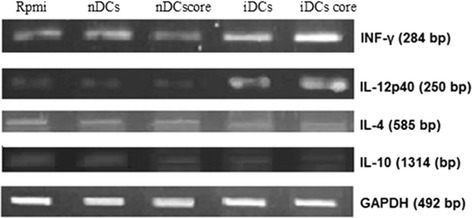
Effect of mice immunization on the expression of splenic INF-γ, IL-12p40, IL-4 and IL-10 respectively. Cytokine profile of splenocytes of mice immunized with BCE-induced-core-pulsed DCs exhibits a great shifting towards up-regulating Th1 cytokine profile and down-regulating Th2 cytokine profile. Specific PCR amplification of reverse-transcribed total RNA (1 μg) from 1 × 10^6^ of splenocytes of mice immunized with different subsets of DCs for 4 weeks

In order to address the possible modulation in the T helper 1 (Th1)/T helper2 (Th2) cytokines gene expression after mice immunization, the difference in mRNA expression of Th1 cytokines (Interferon-gamma (INF-γ) and interleukin (IL) -12p40) and Th2 cytokines (IL-4 and IL-10) were evaluated by RT-PCR analysis. Th1 cytokines gene expression exhibited a noticeable up-regulation (Fig. 2). Levels of INF-γ mRNA increased in iDCs-core group only. IL-12p40 increased in all groups immunized with different subsets of DCs with the highest IL-12p40 gene expression in iDCs-core group when compared to negative control group. On the other hand, mRNA levels of IL-4 exhibited down-regulation in all groups immunized with different subsets of DCs with the lowest level reported at in nDCs group. A dramatic down-regulation in IL-10 was recorded, except for nDCs group that showed an unexpected increase (Fig. 2).

### High protein expression of intrasplenic INF-γ correlates with elevated levels of mRNA Th1 cytokines and reduced mRNA levels of Th2 cytokines

**Fig. 3:**
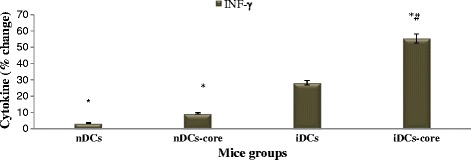
Cytolytic protein expression of INF-γ after mice immunization. Mice immunized with iDCs or iDCs-core cells increases intrasplenic IFN-γ production in their splenocytes when compared to RPMI group and/or nDCs and nDCs-core groups. Cytoplasmic contents of cytokines protein expression of INF-γ was assessed from 1 × 10^6^ of splenocytes using ELISA as previously mentioned at material and methods. Data are shown as the percentage of splenocytes bearing surface antigen over total splenocytes population. * indicates a significant difference with RPMI- 1640 group. Results are means ± SD of splenocytes of six mice. # indicates a significant difference within nDCs, and nDCs-core groups

mRNA level of INF-γ was verified by evaluating protein expression of INF-γ using ELISA (Fig. 3). In vivo immunization of BALB/c mice with different subsets of DCs, resulted in an increase of protein levels of INF-γ in iDCs and iDCs-core groups by 22 % and 47.7 %, respectively (p < 0.05, n = 6).

### Mice group that received BCE-induced-core-pulsed DCs had a good T lymphocyte suppression capacity only when co-cultured with EL4-core cells

A recent study reported that myeloid DCs have an up-regulated cytotoxic activity to kill T cells during HCV chronic infection, which represent a novel mechanism of HCV evasion [22]. EL4 cells (ATCC® TIB39 ™) is a lymphoma cell line induced in a C57BL mouse by 9,10-dimethyl-1,2-benzanthracene.

**Fig. 4:**
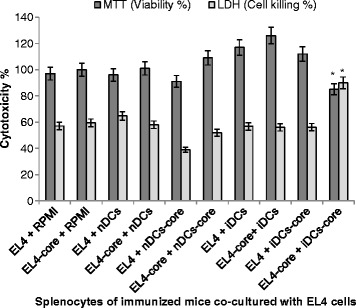
Splenocytes of iDCs-core group have a good T lymphocyte suppression capacity only on EL4-core cells. Splenocytes of immunized mice (E) were co-cultured with EL-4 or EL-4-core cells (T) at a ratio of 1:1.6 E:T. EL-4 cells were transduced with core recombinant peptides using BioPORTER quickEase protein delivery Kit as previously described. Co-cultured cells were incubated at 37 °C for 4 h and their cytotoxicity was assessed using MTT viability test and LDH release. Data are shown as the percentage of splenocytes bearing surface antigen over total splenocytes population. * indicates a significant difference with RPMI group. Results are means ± SD of splenocytes of six mice. # indicates a significant difference within nDCs, and nDCs-core groups

To investigate the anti-core T lymphocyte activity of immunized mice’s splenocytes, EL4 target cells (T) were co-cultured with splenocyte effector cells (E) of immunized mice [24]. The optimum co-culture ratio of E:T cells was 1:1.6 and determined by measuring cytotoxicity of the co-culture of splenocytes of non-treated mice and murine EL4 lymphoma cells using MTT cell viability assay. Cytotoxicity of EL4 cells (transduced and non-transduced) co-cultured with splenocytes of immunized mice was represented by the percentages of cell viability (MTT assay), and cell killing (LDH leakage). Cell viability of iDCs-core mice group significantly decreased (p < 0.05) by 12.4 % when co-cultured with EL4-core cells (Fig. 4)., This was associated with increased cell killing (57.9 %), in comparison with EL4 + RPMI - 1640 However, the rest of mice groups had no significant differences (p > 0.05) to each other.

### Potent and specific anti-core IgG antibodies only in the group that was immunized with BCE-induced-core-pulsed DCs

**Fig. 5:**
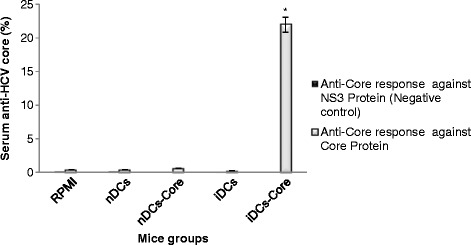
Antigen-specific IgG antibodies generated in mice by immunization with DCs. Humoral response assessment revealed potent and specific anti-core IgG antibodies (total IgG) only in iDCs-core group. Detection of igG antibody responses to the HCV core protein in immunized mice by ELISA. Pooled sera (1:50 dilution) from each group were incubated in wells coated with recombinant HCV core protein (NS3 protein was used as a negative control) and detected by a goat anti-mouse secondary antibody (immuno- globulin) conjugated to alkaline phosphatase. The results are expressed as the optical densities (O.D.) at 405 nm. * indicates a significant difference among groups. Results are means ± SD of sera of six mice

Anti-HCV core IgG antibodies levels in the serum of each immunized mouse were monitored to assess the humoral immune response of mice to injection with DCs. Anti-core IgG antibodies were detected in the sera of iDCs-core group only when incubated with core recombinant protein at a percentage of 21.9697 % after one week of the last subcutaneous boosting dose (p < 0.05, n = 6). No antibodies were detected when iDCs-core sera when incubated with NS3 protein. On the other hand, a minor percentage of antibodies against core and NS3 proteins was detected in groups of RPMI - 1640 and nDCs, but did not reach the value of a positive specific anti-core IgG (Fig. 5).

## Discussion

### Impaired cellular functions of immune system upon HCV

Impairment of the maturation process in dendritic cells (DCs) is one of the mechanisms responsible for immune evasion of hepatitis C virus (HCV) [6]. A study reported that myeloid DCs have an up-regulated cytotoxic activity to kill T cells during HCV chronic infection, which represents a novel mechanism of HCV evasion [22]. HCV core protein can interact with multiple cellular factors, and regulate expression of cellular genes and control signaling pathways of different cell types, including DCs [25, 26].

Several studies have shown that core protein may have a pro-apoptotic activity by affecting the tumor necrosis factor receptor (TNFR) signaling pathway. Since lymphotoxin β receptor (LTβR) is involved in apoptotic signaling, this suggests that core protein may have an immunomodulatory function and may also play a critical role in the establishment of HCV persistence and in disease pathogenesis [26, 27]. Therefore, the cytoplasmic domain of LTβR and TNFR1 signaling is important for the microenvironment that allows interactions of lymphocytes with antigen presenting cells and for B cell migration and differentiation into antibody-producing cells. Impairment of these functions may interfere with the elimination of infected cells and neutralization of virus. [28] Core protein reduces sensitivity to tumor necrosis factor (TNF) and activates nuclear factor kappa B (NFkB), thus inhibiting apoptosis either constitutively or in response to cytokines [29]. HCV can infect T cells, so any decrease in the apoptosis thresholds by core may impair their activation and cytotoxic functions. Since HCV can infect lymphocytes, increasing their sensitivity to apoptotic stimuli affects their activation and immune functions. In chronic infection, few HCV specific cytotoxic T lymphocytes (CTL) are found in the peripheral blood, which could be due to an abnormal death of activated CTL [30, 31].

Interaction of virus particle with cell surface molecules, such as CD81, may modulate cell signaling. CD81 is widely expressed and found on natural killer (NK) cells. Inhibition of interferon-gamma (INF-γ) production by NK cells could alter the development of a T helper 1 (Th1) response and favor a T helper 2 (Th2). Inhibition of the innate immune response early after infection could confer a growth advantage to HCV that could not be controlled by the adaptive immune response. The inefficient NK cell response could allow the selection of escape variants [32]. Compared to monocyte-derived DCs from healthy donors, DCs from patients with chronic HCV infection showed an impaired ability to stimulate allogeneic T cells and to produce interferon (INF) [33]. This impaired maturation of DCs has been correlated with persistent HCV infection, [34, 35] and that’s the main scope of the current study.

### Role of BCE on DCs enrichment

A number of studies described a significant down-regulation of DCs function in HCV infected patients, shifting the Th1/Th2 balance towards Th2 up-regulation [7], so a therapeutic DCs vaccine may hold its promises by ex vivo maturation and stimulation of DCs, because DCs of chronically infected patients are under a negative regulation of the virus itself. It was demonstrated that treatment of macrophages and DCs with berberine; a benzodioxoloquinolizine alkaloid present in *Berberis vulgaris* plant, significantly induced interleukin (IL) -12 production in a dose-dependent manner, leading to the inhibition of Th2 cytokine profile in CD4^+^ T cells which was also observed in our results (Table 1) with a decrease in Th1 cytokines (IL 10, and IL4) in both protein and mRNA level [13–15, 17–36] (https://dragonherbarium.com/products/barberry-root-bark-c-s-wc-berberis-vulgaris). In the present study, results of phenotypic characterization of immunized mice’s splenocytes proved that barberry crude extract (BCE) was the most powerful inducer for splenic cells to express DCs surface markers with acquiring the functional characteristics associated with DCs. The maximal increase CD11c^+^ cells of animal model (A) after BCE intravenous injection by 10 folds with an increase in MHC II surface marker which indicate an increase in antigen presenting capacity (Table 1).

**Table 1:** Phenotyping analysis of DCs

Parameters	BCE-induced-DCs	Non-BCE-induced-DCs
CD11c (%)	16 ± 0.9	1.5 ± 0.01
MHCII (%)	13 ± 0.4	8 ± 0.3
IL4 (fold)	0.31 ± 0.01	0.61 ± 0.002
IL 10 (fold)	0.27 ± 0.03	0.73 ± 0.002
IL 12 (fold)	0.74 ± 0.005	0.2 ± 0.003
INF (fold)	1.3 ± 0.05	0.56 ± 0.002
IL 12 (Pg/ml)	4.5 ± 0.3	1.2 ± 0.1
INF-gamma (Pg/ml)	35 ± 3.2	22.5 ± 1.2

The level of CD11c and MHCII were detected by FACSCalibur flow cytometer (BD Biosciences). cytokines expression (IL4, IL10, IL 12 and INF-gamma) was performed by RT-PCR. IL 12 and INF-gamma proteins levels were measured by ELISA (Komabiotech, Korea)

CD11c increase in DCs treated with BCE had exceeded the conventional DCs stimulators such as recombinant granulocyte-macrophage colony-stimulating factor (GM-CSF) and recombinant fms-like tyrosine kinase receptor-3 (Flt3) ligand according to a study by Berhanu el al. [37] These workers found that BALB/c mice subcutaneous injection with GM-CSF had expanded splenic CD11c^+^ cells by about one fold, and Flt3 ligand injection expanded them by about five folds, whereas the combinatory GM-CSF and Flt3 ligand injection had expanded CD11c^+^ cells by 9 folds.

CD16-mediated signal transduction promotes induction of maturation of immature DCs and CD16-mediated antigen uptake potently enhances antigen presentation [20, 21]. CD16^+^ cells in iDCs-core splenocytes exceeding those in RPMI - 1640 group by approximately 3 folds, reflected an up-regulation in pro-inflammatory cytokines that might represent DCs precursors. Moreover, it might indicate an elevated phagocytic activity and antigen presenting capacity of the same group [38]. Elevated expression of major histocompatibility (MHC) II surface antigen on splenocytes of iDCs-core group identified a high regulation of antigen presentation, DCs morphology, and expression of co-stimulatory molecules which was supported by Banchereau, et al. [22] Although CD16 is not DCs specific, it was found on a limited number of other cell types like NK cells and macrophages [39]. However, co-expression of CD16 with MHC-II has been previously described by Haverson [40] as being characteristic for DCs in the porcine intestinal lamina propria. While another important marker for splenic DCs is CD11c, since the injected cells used for immunization were solely CD11c^+^. Low CD3^+^ cells from immunized mice may be attributed to the architectural changes in the T cell receptor (TCR):CD3 complexes induced by MHCII:core protein ligation after immunization with DCs induced by BCE and pulsed with core protein (as MHCII T cell interaction complex causes internalization and degradation of CD3 antigen). The rate of TCR internalization did not increase significantly, while the binding of a peptide: MHCII ligand to the TCR promoted significant down-regulation of TCR complexes. This trend suggested that the binding of the ligand blocks TCR complexes from returning to the cell surface rather than increasing the rate of internalization of TCR complexes expressed on the cell surface [41].

The present phenotypic reports clearly emphasized the important role BCE on DCs enrichment and maturation. It also emphasized that immunization with BCE-induced DCs might counteract DCs defects that took place upon HCV infection.

### Vaccine-mediated immune response

HCV core protein triggered monocyte-derived IL-10 productions. This cytokine, in turn, has led to DCs apoptosis and impaired production of interferon-alpha (INF-α), thus closely resembling the DCs defects seen in chronically HCV-infected patient. Multiple immune defects have been described in patients with chronic HCV infection that might be linked to the reduced INF-α production, including insufficient response of CTL, low activity of NK cells, and production of antibody with low neutralizing capacity [42, 43]. Most antigens and vaccines trigger both B and T cell responses, such that there is no rationale in opposing antibody production (humoral immunity) and T cell responses (cellular immunity). In addition, Th cells are required for most antibody responses, while antibodies exert significant influence on T cell responses to intracellular pathogen [44, 45]. Vaccine–induced immune effectors are essentially antibodies produced by B lymphocytes and capable of binding specifically to a toxin or a pathogen peptide. Other potential effectors are CTL that may limit the spread of infectious agents by recognizing and killing infected cells or secreting specific antiviral cytokines. The generation and maintenance of both B and CTL responses is supported by growth factors and signals provided by Th cells. These effectors are controlled by regulatory T cells that are involved in maintaining immune tolerance.

### Anti-core cellular response

The communication between DCs and T cells in this area of host–pathogen interaction seems to be a dialogue rather than a monologue in which mature DCs respond to T cells as well. In the process of studying DCs vaccine, it is worth mentioning that INF-γ, IL-12, IL-4, IL-10, and other cytokines had a crucial role in manipulating a potent anti-HCV cellular immune response after vaccination. Up-regulation of Th1 cytokines (e.g. IL-12, INF-γ) and down-regulation of Th2 cytokines (e.g. IL-4, IL-10) and nitric oxide (NO) were the major targets to retain the th1/th2 balance corrupted by HCV viral infection and have an adequate anti-HCV cellular immune response. Interferons are key players of the innate immune response to virus infection. The production of INF-γ (type II INF) is restricted to cells of the immune system, such as NK cells, macrophages, and T cells [46]. IL-12, which is known to be the primary stimulator of NK cells and INF-γ, induce Th1 type cytokines and foster CTL development. IL-12 is, therefore, critical in a series of immune-pathological conditions, such as viral infections and tumors [47, 48]. On the other hand, IL-4 exists in elevated levels in sera of HCV infected patients than non-infected ones [49]. It is known to down-regulate cell-mediated immune effector mechanisms important in the host defense against intracellular pathogens [50]. IL-10 is a pleiotropic cytokine traditionally considered as immunosuppressive and anti-inflammatory, produced by many cell types, [51, 52] which exerts its effects by inhibiting macrophage and DCs functions. In chronic HCV infection, patients have high serum levels of IL-10, associated with incomplete responses to INF therapy [53].

RT-PCR analysis of immunized mice splenocytes m-RNA demonstrated an increase in mRNA expression of IL-12p40 and INF-γ transcripts in iDCs-core, and iDCs groups (Fig. 2), indicating a potent up-regulation in Th1 arm. While a down-regulation in mRNA expression in IL-10 transcripts was demonstrated at all groups that were immunized with different subsets of DCs with the lowest IL-10 mRNA expression in case of iDCs-core group (Fig. 2), indicating a down-regulation in Th2 arm. Protein expression of intrasplenic INF-γ, (the target protein) was high and in accordance with its mRNA expression (Fig. 3). This ascertains that the immune system is having a better struggle with the viral infection to prevent its persistency. On the other hand, protein expression of IL-12 was unexpectedly diminished. One mechanism that might account for the low intra-splenic IL-12 protein level was that it might be consumed by the cells [54]. Alternatively, IL-12 has a half-life of only 12 h), [55] or may have released out of the cell and functioned extracellularly. This postulate was assisted by the up-regulation of splenic IL-12p40 mRNA expression.

In the present study, the strongly reduced extracellular levels of blood NO of all immunized mice with different DCs subsets, with the lowest NO level in iDCs-core group, was indicative for a selective and persistent up-regulation of T cells INF-γ production. This finding was supported by Roozendaal et al. [56] where NO was able to modulate the balance between the expression of Th1- and Th2-type cytokines through selective and persistent inhibition of expression of INF, shifting Th1/Th2 balance towards Th2 cytokines. It is worth noting that the main function of this vaccine was to counteract the effect of HCV infection of misbalancing the Th1/Th2 model.

CTL assay was performed to determine and quantitate the capacity of effector pre-induced cells (splenocytes of nDCs-core, iDCs-core groups individually), to eliminate target cells (EL4-core cells). The principle of this assay was to prime T cells to be antigen-specific through the immunization process and then, upon repeated exposure to a specific antigen, induce a rapid T cell expansion. The study carried out to determine the best number of co-culture between splenocytes and EL4 proved to be essential to ensure that EL4 and splenocytes were able to be cultivated together, and to determine the optimum effector:target cells ratio with the highest viability to use it for T lymphocyte suppression assay. CTL assay showed that splenocytes of iDCs-core group had a good T lymphocyte suppression capacity only on EL4-core cells (Fig. 4), indicating a specific T response to the core-pulsed cells. In this experiment, DCs have been successfully used as cellular adjuvants in mice to elicit protective T cell immunity against HCV core protein.

### Anti-core humoral response

To determine if adequate levels of antibodies had been attained following vaccination, IgG antibodies recognizing HCV core protein were measured. Humoral response assessment revealed specific anti-core IgG antibodies only in iDCs-core group, with a remarkable high percentage against negative control (Fig. 5). This result reflects a significant B cells activation to produce specific antibodies against core protein. Interestingly, iDCs-core group that demonstrated the most significant humoral response also showed the highest cellular immune response.

In conclusion, the present study provides evidence for the role of BCE on stimulating splenic DCs proliferation, on both sides of DCs maturation and count. In addition, the basics for a novel vaccination strategy to treat HCV were manipulated, through analyzing the humoral and cellular immune response upon immunizing mice with different subsets of treated DCs. Therapeutic efficacy of BCE synergized core-pulsed DCs vaccination exhibited an interesting anti-core boosting effect against a tumor model expressing HCV core protein in an immunocompetent host (EL4-core cells). DCs are essential for T cells activation and since viral clearance in HCV infected patients is associated with a vigorous T cells response, a new type of HCV vaccine of a strong antigenicity in humoral and cellular immunities was proposed, based on ex-vivo stimulated HCV core protein-pulsed DCs and promoted by BCE treatment. This vaccine might have a therapeutic setting to potentially circumvent the diminished and down-regulated DCs function of HCV infected patients by giving the necessary maturation stimuli whether in vivo (BCE treatment) or in ex vivo (core protein transduction). As a therapeutic immunization approach, this vaccine may pertain new aspects compared to other vaccines, which are faced in HCV infected patients with down-regulated DCs function.

It is crucial that further studies should be carried out to reliably move this preclinical study to a clinical one. The ultimate goal in the near future is to conduct some research on the CD4 and CD8 T responses, and IL-28b monitoring, in order to full scheme the therapeutic influence of the proposed vaccine. Working on peripheral blood of HCV infected patients should be included. Finally, further clinical studies should be carried out on pegylated interferon non-responders HCV patients with low doses of vaccine tailored from each patient’s blood, for tracking its therapeutic effect.

## Conclusion

Because DCs are essential for T cell activation and since viral clearance in HCV infected patients is associated with a vigorous T cell response, this study proposed a new type of therapeutic HCV vaccine model of a strong antigenicity in humoral and cellular immunities, based on ex vivo stimulated HCV core protein-pulsed DCs and promoted by BCE treatment. This model might be used in a therapeutic setting to circumvent the diminished and down-regulated DCs function of HCV infected patients. As a therapeutic immunization approach this vaccine may present important new aspects since other vaccines are faced in HCV infected patients with the described down-regulated DCs function.

## Abbreviations

BCE: Barberry ethanolic crude extract
BSA: Bovine serum albumin
CD: Cluster of differentiation
CTL: Cytotoxic T lymphocytes
DC: Dendritic cells
ELISA: Enzyme linked immunosorbent assay
Flt3: Fms-like tyrosine kinase receptor-3
GM-CSF: Granulocyte-macrophage colony-stimulating factor
HCV: Hepatitis C virus
iDCs group: That received BCE-induced DCs
iDCs-core group: That received BCE-induced DCs transduced with core protein
IFN-α: Interferon α
IL 12: Interleukin 12
LDH: Lactate dehydrogenase
LTβR: Lymphotoxin β receptor
MHCII: Major histocompatibility complex class II
MTT assay: 3-(4,5-Dimethylthiazol-2-yl)-2,5-diphenyltetrazolium bromide assay
nDCs group: That received non-BCE-induced DC
nDCs-core group: That received non-BCE-induced DCs transduced with core protein
NFkB: Nuclear factor kappa B
NK cells: Natural killer cells
NO: Nitric oxide
PBS: Phosphate buffered saline
PRPMI - 1640 group: That received complete medium
SVR: Sustained virologic response
TCR: T cell complex receptor
Th1: T helper 1
Th2: T helper 2
TNFR: Tumor necrosis factor receptor
